# Contrasting Strategies in Bouveret’s Syndrome: A Series of Two Cases

**DOI:** 10.7759/cureus.28880

**Published:** 2022-09-07

**Authors:** Andrew J Hsu, Bixuan Lin, Bashar Attar, Benjamin Go

**Affiliations:** 1 Gastroenterology and Hepatology, John H. Stroger, Jr. Hospital of Cook County, Chicago, USA; 2 Gastroenterology and Hepatology, Rush University Medical Center, Chicago, USA

**Keywords:** endoscopic dilation therapy, interventional gastroenterology, endoscopic management, gallstone disease (gsd), gallstone extraction, bouveret's syndrome

## Abstract

Bouveret’s syndrome is a rare complication of cholelithiasis, characterized by gastric outlet obstruction caused by a migrated gallstone. Diagnosis of Bouveret’s syndrome necessitates urgent treatment as it carries a high mortality rate. The treatment of Bouveret’s syndrome has traditionally been surgical. However, there have been increasing reports of successful endoscopic therapy for Bouveret’s syndrome. This case series aims to compare and contrast two cases of Bouveret’s syndrome. The gallstone was retrieved via endoscopic access in one case while the other was removed with surgery. For each case, we discuss the various factors that contributed to the decision of which treatment modality to use. In addition, we propose an endoscopic technique that may improve the safety and success rate of endoscopic treatment of Bouveret’s syndrome.

## Introduction

Gallstone ileus refers to the mechanical obstruction within the gastrointestinal tract by an impacted gallstone and is a rare condition that happens to about 0.3-0.5% of patients with cholelithiasis [1]. Bouveret’s syndrome is a rare variant of gallstone ileus in which a large gallstone, typically >2.5 cm [2,3], compresses against the pylorus or proximal duodenum and causes a gastric outlet obstruction via a cholecystoenteric or cholecystogastric fistula. Among gallstone ileus fistulas, the majority (68%) are cholecystoenteric. The remainder are cholecystocolic and cholecystogastric, which represent 17% and 5% of gallstone fistulas, respectively [4]. Bouveret’s syndrome is associated with a high mortality rate of 12-30%, likely in part due to the advanced age of afflicted patients (median 74.1 years) [4] and associated comorbidities. Successful treatment of Bouveret’s syndrome requires a multidisciplinary approach as well as a thorough knowledge of the available treatment options and patient-specific factors. We describe two such cases of Bouveret’s syndrome wherein the risk and benefit analyses resulted in considerably different strategies of management.

## Case presentation

Case one

A 65-year-old male with a history of chronic cholecystitis, squamous cell carcinoma of the palate status post-chemotherapy, and radiation three years prior followed by tracheostomy and percutaneous gastrostomy placement the following year presented with a five-day history of nausea and vomiting of his tube feeds. The patient also reported a two-day history of chills without fever and mild right lower abdominal quadrant pain and tenderness. On arrival, his vitals were normal including temperature of 36.7°C. On examination, the gastrostomy site in the left upper quadrant of the abdomen appeared unremarkable, and the gastrostomy tube was in place. The patient had mild tenderness over the right lower quadrant of the abdomen. Bowel sounds were normal. Laboratory analysis revealed metabolic derangements, including hyponatremia (130 mEq/L) and hypokalemia (3.4 mEq/L), normocytic anemia (11.3 g/dL), no leukocytosis, and normal liver profile.

**Figure 1:**
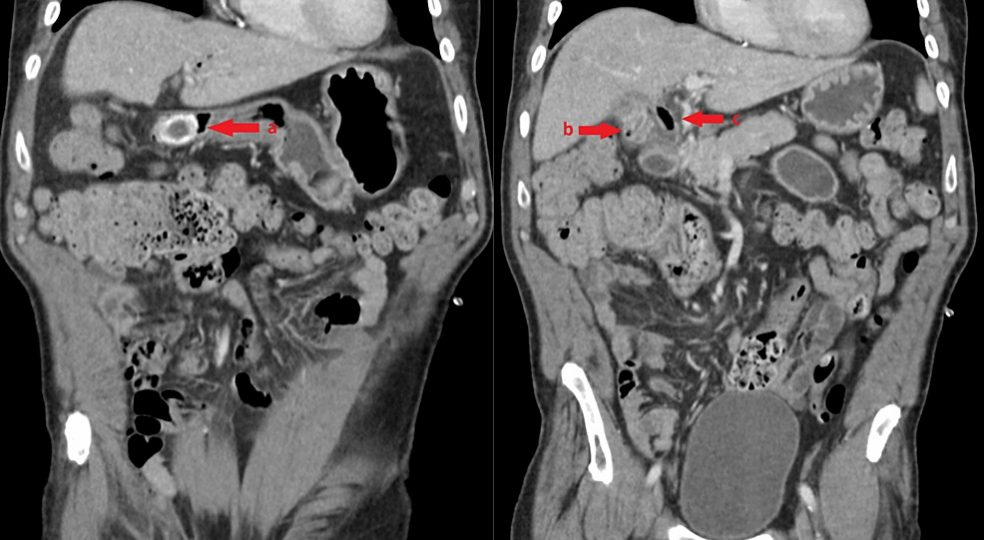
Coronal cross-section of the abdomen and pelvis. Coronal cross-sections of the patient’s abdomen and pelvis demonstrate (a) a dense, calcified gallstone measuring 3 cm located in the proximal duodenum. (b) A decompressed gallbladder with new pneumobilia. (c) Pneumobilia in the common hepatic duct and intrahepatic ducts.

Computed tomography (CT) of the abdomen with intravenous (IV) contrast showed a 3 cm calcified gallstone, which was seen within the gallbladder on previous imaging, in the duodenal bulb along with new pneumobilia (Figure 1). The gallbladder was filled with fluid and not distended. Though suspected, there was no clearly visible cholecystoduodenal fistula. The stomach was not distended, and the G-tube was positioned appropriately in the stomach.

**Figure 2:**
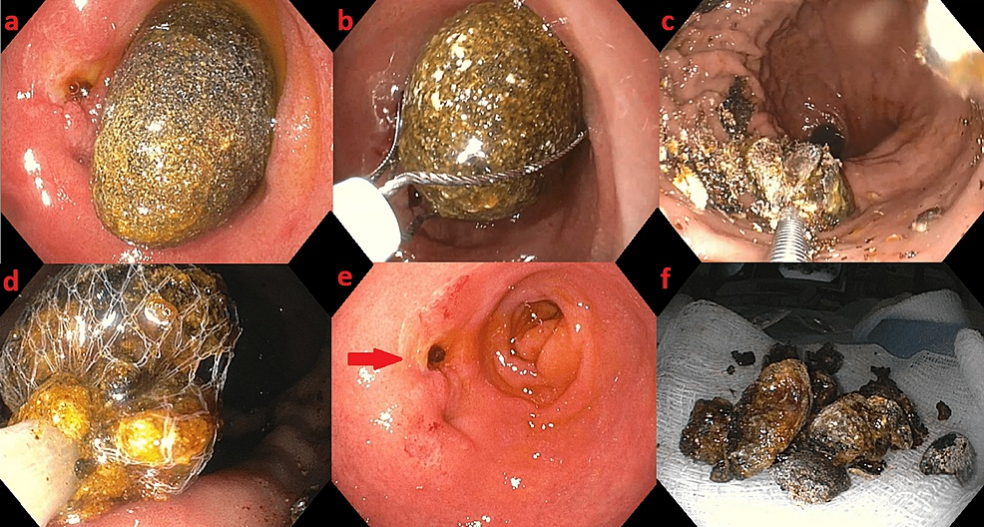
Upper endoscopy. Upper endoscopy demonstrates (a) a large gallstone present in the duodenal bulb, where a small opening is seen draining bilious fluid, consistent with a cholecystoduodenal fistula. (b) The stone is grasped with a 3.5 cm mechanical lithotripsy wire basket and then pulled into the gastric body. (c) The stone is fragmented with the mechanical lithotripsy device. (d) The fragments are successfully removed with a Roth net. (e) The final inspection of the duodenum again shows an ulcerated orifice draining bile. (f) Final specimens after removal.

Due to concern for Bouveret’s syndrome, the patient underwent an upper endoscopy on hospital day two, which showed a large gallstone in the duodenal bulb and a small opening in the adjacent that was draining bilious fluid, consistent with a cholecystoduodenal fistula (Figure 2). The gallstone was successfully grasped with a 3.5 cm mechanical lithotripsy wire basket. The gallstone was pulled into the pylorus, where it did not readily pass into the stomach. Extraction into the stomach was achieved with two minutes of constant traction. Once the gallstone was in the stomach, it was brought to the mid body where four rounds of mechanical lithotripsy were performed. The fragments were then retrieved sequentially with three Roth nets and removed from the patient. The final inspection revealed a patent duodenal bulb and clean based ulcerations around the cholecystoduodenal fistula. There was no trauma to the pylorus and minimal trauma to the esophagus post-intervention.

The patient was discharged the next day without any apparent complications and with complete resolution of his symptoms. Cholecystectomy was not pursued due to his multiple comorbidities, as well as the lack of any residual gallstones in the gallbladder based on initial CT imaging.

Case two

A 64-year-old male with a medical history of obesity, hypertension, and low-grade breast phyllodes tumor presented with a two-day history of sudden-onset epigastric pain radiating to the back and bilateral flanks. His symptoms were associated with hematemesis, followed by coffee ground emesis. He had a similar but less severe episode of epigastric pain three months prior, for which he did not seek medical attention. Initial vital signs were normal. His abdomen was soft but moderately distended with tenderness over the epigastrium and right upper abdominal quadrant areas. There was no rebound tenderness, and bowel sounds were normal. Laboratory findings included a white blood cell count of 17,400/μL. Complete blood count and basic metabolic panel were normal.

**Figure 3:**
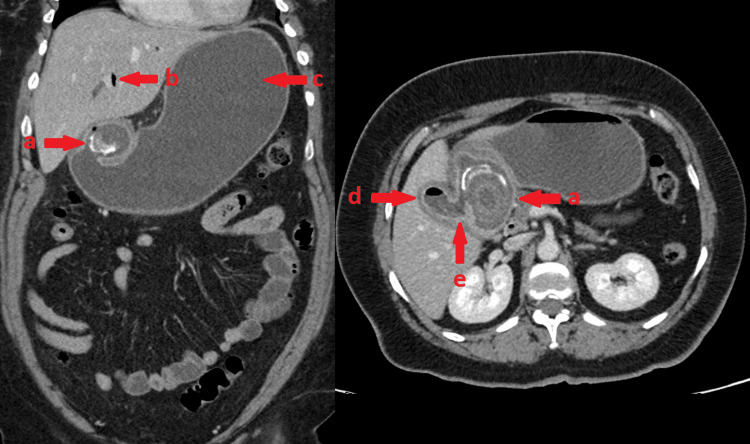
Coronal and axial cross-sections of the abdomen and pelvis. Coronal and axial cross-sections of the patient’s abdomen and pelvis demonstrate (a) a calcified gallstone present in the duodenal bulb measuring 6 cm in the maximal dimension. (b) Pneumobilia. (c) A fluid-filled and distended stomach. (d) An air fluid level in the non-distended gallbladder. (e) A fistulous connection between the gallbladder and the adjacent duodenum.

CT of the abdomen with IV contrast showed a gastric outlet obstruction secondary to a 6 cm × 3.5 cm obstructing calcified gallstone in the duodenal bulb. Air and fluid were present in a non-distended gallbladder and a cholecystoduodenal fistula was seen, consistent with a diagnosis of Bouveret’s syndrome (Figure 3).

Because the stone size exceeded the size of the largest wire basket available at the hospital, mechanical lithotripsy was not feasible. Electrohydraulic lithotripsy and laser lithotripsy were also not readily available. Thus, the patient proceeded with surgical management. Exploratory laparotomy on hospital day three revealed that the gallstone seen on imaging was palpable within the duodenal bulb and pyloric channel. The stone was manually maneuvered into the stomach, and a gastrostomy was performed to remove the stone. After successful stone removal, the gastrostomy was closed. The gallbladder appeared chronically inflamed and adherent to the adjacent liver and duodenum. No further intervention was pursued. The abdomen was then closed. Postoperatively, the patient developed bacterial pneumonia, but then quickly recovered after initiating antibiotics. The patient was discharged home on postoperative day seven.

## Discussion

Bouveret’s syndrome is a heterogeneous clinical entity owing to variability in presenting factors. By definition, a gastric outlet obstruction is caused by the migration of a gallstone through a cholecystoduodenal or cholecystogastric fistula. Symptoms vary depending on the degree of obstruction, location of the stone, and other complicating factors. Common symptoms include intermittent nausea and vomiting in 85% of the cases and abdominal distension and pain in 70% of the cases. Patients may present with dehydration (31%), weight loss (14%), hematemesis (15%), and, rarely, the expulsion of the stone in the vomitus. Patients often present to the hospital five to seven days after the onset of symptoms. Obstructive jaundice is seen in 33% of patients. Examination and laboratory findings tend to be non-specific [5].

The clinical diagnosis is established once imaging or direct visualization confirms the presence of an obstructing gallstone in the gastric outlet. A plain film of the abdomen may be diagnostic in as many as 21% of cases with classic findings of pneumobilia, intestinal obstruction, and an aberrantly located gallstone along with air-fluid levels within the gallbladder [6]. A review of prior imaging may demonstrate a migrating stone as the fistula evolves. More often patients are diagnosed on cross-sectional CT, which is 60-93% sensitive and 100% specific for the disease; it can also help assess for intra-abdominal complications and rule out other differential diagnoses [5,6]. For patients lacking a clear etiology for their gastric outlet obstruction, upper endoscopy can be diagnostic with visualization of the gallstone in up to 69% of cases, though the cause of the gastric outlet obstruction may not be apparent if the gallstone has not completely migrated into the enteric lumen, in which case it may be deeply embedded within the mucosa [5,7].

Multiple case reports and series suggest that upper endoscopy should be the first line in the evaluation and treatment of Bouveret’s syndrome. While a case series from 2007 reported a limited success rate of 10% [5], recent literature includes numerous reports of successful endoscopic management of the disease. Endoscopic management has the potential to eliminate the need for invasive surgical procedures, which is often desirable in the context of Bouveret’s syndrome, as the median presenting age is 74 years old [4] and the reported postsurgical mortality is 12-24% [8]. Some patients may have already forgone surgical intervention for their known cholelithiasis due to comorbidities, such as in Case one. The size of the stone should also be considered. Gallstones in Bouveret’s typically range between 2 cm and 8 cm [6]. As the stone size increases, certain options such as mechanical lithotripsy become unfeasible. Thus, the true success rate of endoscopic intervention varies from center to center, depending on the availability of proposed techniques, such as electrohydraulic lithotripsy, laser lithotripsy, and extracorporeal shockwave lithotripsy [7].

Possible iatrogenic complications from attempted stone removal must also be considered prior to endoscopy. Aside from the conventional complications of endoscopy of bleeding and perforation, there are additional risks such as gallstone ileus from stone fragments migrating distally during attempted lithotripsy [9,10]. Large duodenal ulcers are sometimes seen as a result of pressure ischemia from the gallstone or as sequelae of the recent fistulation, and thus patients are at an increased risk of perforation. For these reasons, the ability to move the gallstone from the duodenum into the stomach is favorable both from a technical standpoint and to minimize the risk of complications. This is likely a necessary step as 85% of cases of Bouveret’s syndrome involve an obstructing stone in the duodenum, rather than the stomach [11]. All fragments over 2 cm should be removed from the stomach or further fragmented to avoid small bowel obstruction.

One might assume that balloon dilation of the pylorus is required before attempting stone extraction from the duodenum, but this is not strictly necessary and can be technically difficult given the limited working space of an obstructed duodenum. In our case series, we have demonstrated that in both endoscopic and surgical approaches, simple traction is sufficient to dilate the pylorus and accommodate stone passage into the stomach. The orientation, shape, and size of the stone lend themselves ideally to pyloric dilation. In Case one, dilation of the pylorus with stone traction resulted in no visible mucosal trauma. In Case two, a stone measuring 3.5 cm × 6 cm was successfully “milked” from the duodenum into the stomach during exploratory laparotomy. Studies in other clinical scenarios have established that a healthy pylorus can accommodate dilation to 30-35 mm [12], though care should be taken to assess for strictures prior to attempting the maneuver.

In contrast to endoscopy, the success rate of surgery is reported to be nearly 90%, though the associated postoperative mortality ranges from 19% to 24% [13]. There are numerous strategies proposed, with the preferred being a two-stage approach of enterolithotomy or gastrotomy, followed by cholecystectomy and fistula closure. This is favored as fistula takedown at the time of presentation is technically difficult and often unnecessary due to the high rates of spontaneous fistula resolution of up to 61.5% [14].

The complexity and heterogeneity of Bouveret’s syndrome patients demand an individualized approach. Some may be managed with surgery alone, while others benefit from endoscopy. Although endoscopy is generally thought of as low risk, there are complications unique to attempting stone removal in the gastric outlet, particularly for stones impacted in the duodenum. For patients who are good surgical candidates, and for whom endoscopy is unlikely to be successful, forgoing endoscopy may help avoid these complications and expedite the definitive management of their underlying condition.

## Conclusions

The success of endoscopy for the management of Bouveret’s syndrome has increased over the years owing to the use of techniques such as mechanical lithotripsy, electrohydraulic lithotripsy, laser lithotripsy, and extracorporeal shockwave lithotripsy. Because patients with Bouveret’s syndrome tend to be poor surgical candidates due to advanced age and comorbidities, endoscopy is fast becoming the first-line therapy. Despite these advances, however, an individualized approach should still be taken. The choice of endoscopy versus surgery should be made after careful weighing of the risks and benefits, as well as the availability of treatment options. Endoscopic stone removal is often challenging owing to the large size of these stones combined with the limited working space of the duodenum, which is the more common location of gallstone migration when compared to the stomach. We have demonstrated a successful technique for stone removal in a patient with an obstructing duodenal gallstone by moving the stone into the stomach prior to lithotripsy. Pulling the stone under traction is sufficient to dilate the pylorus and allow for stone passage and eliminates the potential risks associated with attempting balloon dilation in a limited working space. This technique may increase the rate of success and minimize the risk of complications. We contrast this case with that of a younger, healthier patient for whom endoscopy was unlikely to be successful due to the size of the gallstone, and thus the surgical approach was the optimal first step in management.
